# Renal-limited thrombotic microangiopathy after switching from bevacizumab to ramucirumab: a case report

**DOI:** 10.1186/s12882-018-1194-9

**Published:** 2019-01-11

**Authors:** Ryo Yamada, Takao Okawa, Ken Matsuo, Makoto Suzuki, Noriko Mori, Kiyoshi Mori

**Affiliations:** 1 Department of Nephrology, Shizuoka General Hospital, Shizuoka, 420-8527 Japan; 2 Department of Pathology, Shizuoka General Hospital, Shizuoka, 420-8527 Japan; 3 Department of Nephrology and Kidney Research, Center for Public Health, Shizuoka General Hospital, Shizuoka, 420-8527 Japan

**Keywords:** Bevacizumab, Nephrotic syndrome, Proteinuria, Ramucirumab, Thrombotic microangiopathy

## Abstract

**Background:**

It is well known that vascular endothelial growth factor (VEGF) inhibitors can cause proteinuria. The incidence of proteinuria is high for bevacizumab, a humanized monoclonal antibody directed against VEGF, but the range of proteinuria rarely becomes nephrotic (2.2% occurrence according to a meta-analysis). In such cases, renal pathology shows thrombotic microangiopathy (TMA). Ramucirumab, anti-VEGF receptor 2 (VEGFR2) monoclonal antibody, can also cause proteinuria, but it is not yet reported whether the drug may induce TMA.

**Case presentation:**

Here, we report a case who immediately developed TMA by ramucirumab after multiple courses of bevacizumab treatment. This is the first case of pathologically-proved TMA by ramucirumab. After cessation of the drug, symptoms of TMA improved gradually.

**Conclusions:**

This case demonstrates that not only blockade of VEGF but also VEGFR2 antagonism may result in TMA, which is a rare but life-threatening complication of cancer treatment drug.

## Background

Vascular endothelial growth factor (VEGF) inhibitors are increasingly applied to treat a number of malignancies such as metastatic or recurrent colorectal, non-small cell lung, breast, and renal cell cancers. Bevacizumab, a humanized monoclonal antibody targeted against VEGF, is broadly used as the first- or second-line therapy to various malignancies [1]. The drug often causes proteinuria in treated patients [2], but it is rare bevacizumab-induced proteinuria reaches to a nephrotic range [3]. In such cases, renal pathology shows thrombotic microangiopathy (TMA) and, at the molecular level, inactivating VEGF signaling leads to damage of glomerular endothelial cells [4]. Other drugs, such as VEGF Trap (anti-VEGF ligand inhibitor) [5] or multi-tyrosine kinase inhibitors (including sunitinib, sorafenib and pazopanib) [6] may cause proteinuria and TMA.

Ramucirumab, anti-VEGF receptor 2 (VEGFR2) monoclonal antibody, is approved for treating gastric, non-small-cell lung, and metastatic colorectal cancers as the second-line therapy. Ramucirumab also potentiates proteinuria [7], but it is unknown whether ramucirumab use is associated with development of TMA. Here, we report a case in which TMA occurred immediately after switching chemotherapy from bevacizumab to ramucirumab.

## Case presentation

**Fig. 1:**
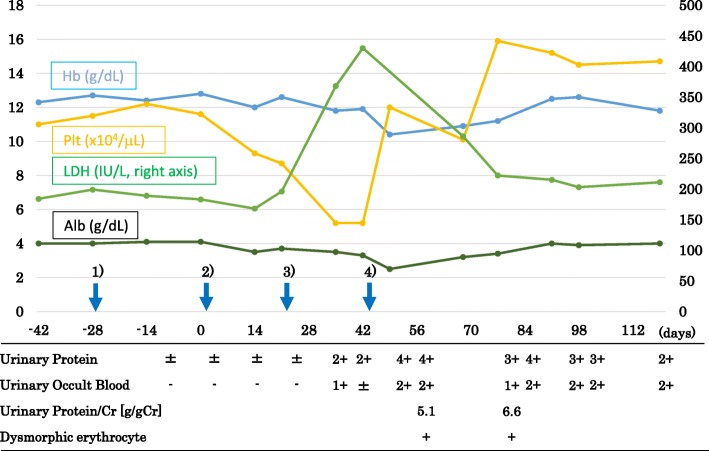
The clinical course of the case. 1) The 23rd administration of the primary therapy (containing bevacizumab) on day − 28. 2) The first administration of the secondary therapy (containing ramucirumab) on day 1. 3) The second administration of the secondary therapy (containing ramucirumab) on day 22. 4) Renal biopsy on day 45

**Fig. 2:**
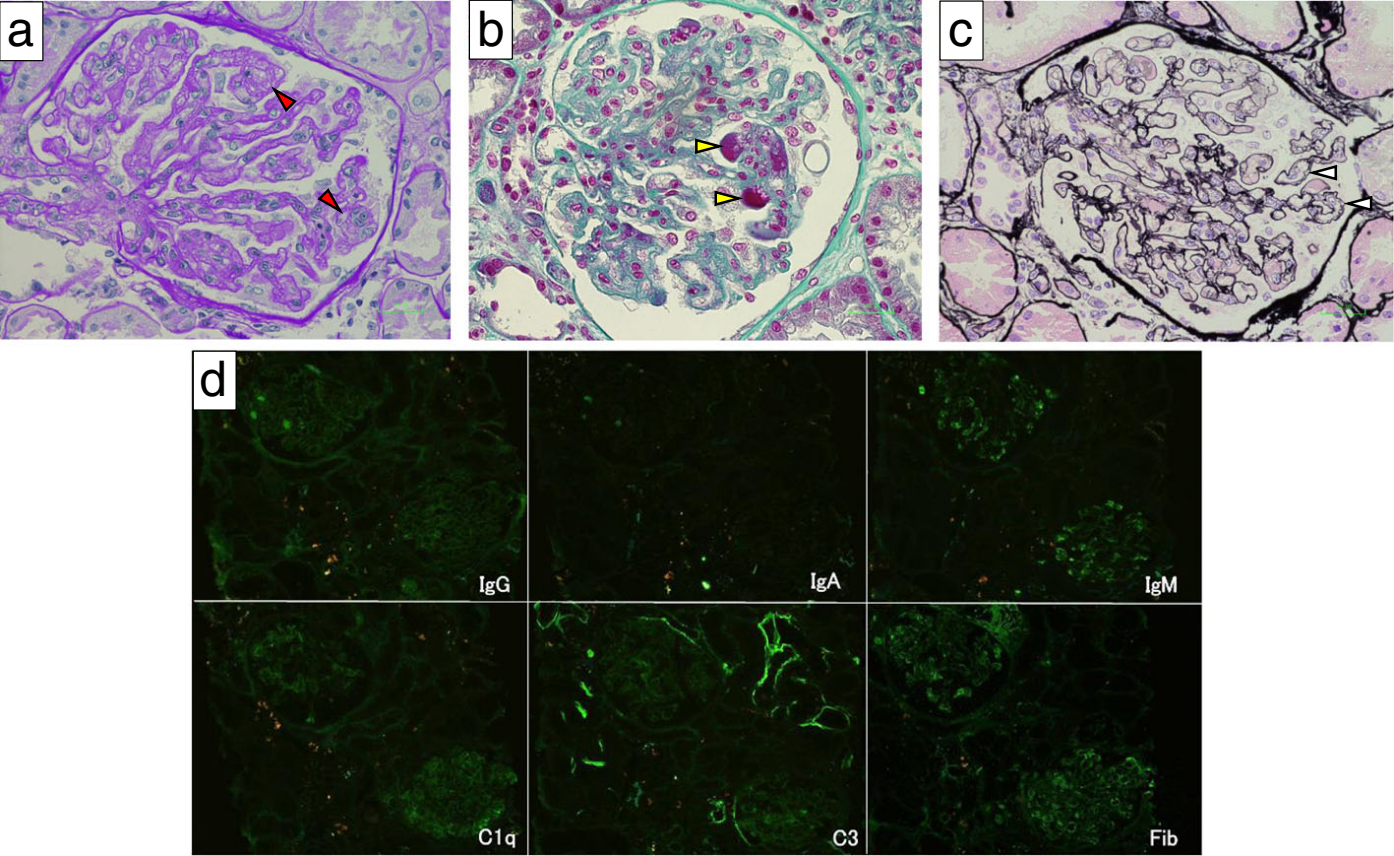
Renal biopsy findings of the case. **a** Periodic acid–Schiff staining showing mesangiolysis (red arrowhead). Original magnification 40X. **b** Azan staining showing fibrin thrombi (yellow arrowhead). 40X. **c** Periodic acid-methenamine silver staining showing double contour of the basement membrane (white arrowhead). 40X. **d** Immunofluorescence for IgG, IgA, IgM, C1q, C3 and fibrinogen (Fib). 20X

**Fig. 3:**
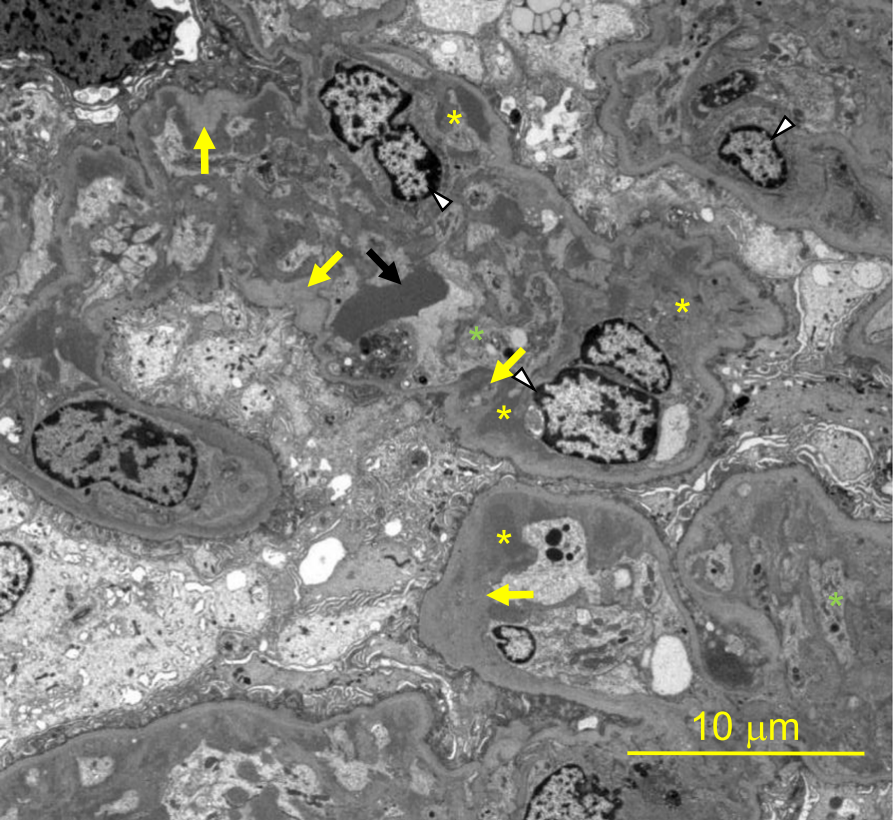
Electron micrograph. Electroscopic imaging showed endothelial cell enlargement (white arrowhead), subendothelial swelling (yellow asterisk), diffuse mesangiolysis and obstruction of the capillary lumen (green asterisk), widening of the glomerular basement membrane (yellow arrow) and deformed erythrocyte (black arrow)

A 75-year-old woman was diagnosed as having stage IV transverse colon cancer in our hospital and began to receive chemotherapy in another hospital. As the first therapy, mFOLFOX6 (modified fluorouracil, leucovorin and oxaliplatin) plus bevacizumab (anti-VEGF-A antibody) was administered for 23 courses over 1 year with few side effects (Fig. 1). Since her cancer status was progressive disease after the above treatment, the secondary therapy, FOLFILI (fluorouracil, leucovorin and irinotecan) plus ramucirumab (anti-VEGFR2 antibody) was started. After 2 courses of therapy (on days 1 and 22), she gradually developed anasarca and nephrotic syndrome and was referred to our nephrology clinic. On immediate admission on day 44, her blood test showed thrombocytopenia (platelets 57,000/μL), slight normocytic anemia (hemoglobin 11.3 g/dL), mild hypoalbuminemia (3.0 g/dL), and mild increase of lactate dehydrogenase level (433 IU/L) (Table 1). Schistocytes were not found on the peripheral blood smear. Haptoglobin level was not measured. Urinary protein was 5.1 g/g-creatinine and dysmorphic erythrocytes were found in the urine. Renal biopsy on day 45 showed fibrin thrombi within capillary loops, mesangiolysis and double contour of the basement membrane of glomeruli (Fig. 2 a, b, c). Immunofluorescence showed weak deposition of IgM, fibrinogen and C1q in mesangio-capillary regions (Fig. 2d). Electron micrograph showed diffuse endothelial swelling with obliteration of capillary lumina (Fig. 3). Pathology was diagnosed as renal-limited TMA. Other causes of nephrotic syndrome, such as focal segmental glomerulosclerosis, membranous nephropathy and membranoproliferative glomerulonephritis, were denied. Ramucirumab was discontinued after the biopsy. By 6 weeks (day 69) after the last ramucirumab injection, thrombocytopenia (101,000/μL) and hypoalbuminemia (3.2 g/dL) showed improvement.

**Table 1:** Laboratory findings upon admission (on day 44)

Blood test				Urine test	
White blood cell	3800/μL	C-reactive protein	0.09 mg/dL	Gravity	1.023
Neutrophil	57.6%	IgG	590 mg/dL	pH	6.0
Lymphocyte	29.3%	IgA	125 mg/dL	Protein	4+
Hemoglobin	11.3 g/dL	IgM	72 mg/dL	Occult blood	3+
Hematocrit	33.7%	Complement 3	109 mg/dL	White blood cell	0 /HPF
Platelet	57,000/μL	Complement 4	18.8 mg/dL	Red blood cell	10–19 /HPF
Total protein	5.7 g/dL	CH50	47 IU/mL	Dysmorphic erythrocyte	+
Albumin	3.0 g/dL	Hepatitis B s antigen	–	Protein	941 mg/dL
Blood urea nitrogen	19 mg/dL	Hepatitis C virus antibody	–	Creatinine	184 mg/dL
Creatinine	0.65 mg/dL				

*HPF* high power field

## Discussion and conclusions

It is well known that VEGF inhibitors, especially bevacizumab, can induce proteinuria and TMA [4]. A meta-analysis comprising 12,268 patients showed that bevacizumab use was associated with 13.3% incidence of all-grade proteinuria [relative risk (RR) 2.8], 2.2% incidence of grade 3 proteinuria (≥3.5 g/24 h, RR 4.8), and 0.8% incidence of nephrotic syndrome [3]. On the other hand, a recent meta-analysis of 4996 subjects revealed that ramucirumab treatment was associated with 9.4% incidence of all-grade proteinuria (RR 3.4), 1.1% incidence of grade 3 proteinuria (RR 8.3), and 0.04% incidence of nephrotic syndrome [7]. These findings suggest that severe renal side effects may be less common in ramucirumab use compared to bevacizumab use.

The underlying mechanism of VEGF inhibitor-induced proteinuria is based upon a fact that a strict amount of VEGF expressed by podocytes is required to maintain the integrity of glomerular endothelial function and glomerular filtration barrier through VEGFR2 expressed upon endothelial cells [3, 4, 8, 9]. The regulation of susceptibility to VEGF inhibition is not understood well, and it is unknown why a patient who tolerated long-term bevacizumab therapy developed TMA soon after switching to ramucirumab.

Double contour of the glomerular basement membrane is regarded as a chronic lesion [10]. Therefore, before administration of ramucirumab, repeated injection of bevacizumab may have already predisposed the glomeruli of the case presented here to develop nephrotic syndrome easily. Indeed, a recent work by our colleagues has shown that doubling in glomerular filtration of albumin causes only mild increase in albuminuria [11].

As to findings by immunofluorescence, the present case showed weak glomerular deposition of IgM, fibrinogen and C1q, consistently with some cases in a previous report of VEGF inhibitor-induced TMA [6]. In that report, most cases showed renal dysfunction, but serum creatinine levels were less than 1 mg/dl in 20% of the cases. Of note, the current case exhibited normal renal function despite having severe occlusion of glomerular capillaries in some glomeruli of renal biopsy samples.

Cancer drug-induced TMA is divided into 2 distinct categories: types I and II [12]. Type I cancer drug-induced TMA develops dose-dependently, typically, at 6 months or later after starting therapy, and is usually permanent and irreversible (which may be caused by mitomycin C, gemcitabine or oxaliplatin). It is usually associated with acute renal failure. The pathological finding is both arteriolar and glomerular capillary thrombosis. On the contrary, type II cancer drug-induced TMA is not dose-related, may occur any time after initiation of and in the course of treatment (1 dose to 29 months later), and has a high likelihood of recovery by stopping the drug (potentially caused by anti-VEGF agents). Glomerular capillary thrombosis lesions are exclusively found [12]. In our case, oxaliplatin was administered as the first therapy. It was considered that TMA was not caused by this drug, since she had relatively minor kidney failure and her renal pathology showed no arteriolar thrombosis. Therefore, we concluded that this case had type II cancer drug-induced TMA due to anti-VEGF agent(s) and it was clinically indicated that the culprit was ramucirumab. To our knowledge, this is the first biopsy report of ramucirumab-induced TMA.

In summary, we present a metastatic colorectal cancer patient who developed TMA after switching from bevacizumab to ramucirumab. Cessation of ramucirumab led to a gradual recovery from TMA. However, we cannot definitively exclude a possibility that previous treatments by bevacizumab and oxaliplatin had a causative or additional role.

## Abbreviations

RR: Relative risk
TMA: Thrombotic microangiopathy
VEGF: Vascular endothelial growth factor
VEGFR2: Vascular endothelial growth factor receptor 2
